# Association of remnant cholesterol with sarcopenia in Korean adults: a nationwide population-based study using data from the KNHANES

**DOI:** 10.3389/fendo.2024.1391733

**Published:** 2024-08-23

**Authors:** Soo Yeon Jang, Soon-Young Hwang, Ahreum Jang, Kyeong Jin Kim, Ji Hee Yu, Nam Hoon Kim, Hye Jin Yoo, Nan Hee Kim, Sei Hyun Baik, Kyung Mook Choi

**Affiliations:** ^1^ Division of Endocrinology and Metabolism, Department of Internal Medicine, Korea University College of Medicine, Seoul, Republic of Korea; ^2^ Department of Biostatistics, Korea University College of Medicine, Seoul, Republic of Korea

**Keywords:** remnant cholesterol, dyslipidemia, sarcopenia, muscle mass, triglyceride-rich lipoproteins

## Abstract

**Background:**

Mounting evidence indicates the importance of the interplay between skeletal muscles and lipid metabolism. Remnant cholesterol (remnant-C) is considered one of the principal residual risk factors for cardiovascular disease and metabolic disorders; however, there are limited studies on the impact of remnant-C on sarcopenia.

**Methods:**

Data from the Korea National Health and Nutrition Examination Surveys (KNHANES) between 2008 and 2011 were used in this nationwide population-based study. In total, 17,408 participants were enrolled in this study. The subjects were categorized into four groups according to the quartile of remnant-C values. We conducted multivariable logistic regression analysis to evaluate the association between remnant-C and muscle mass measured using dual-energy X-ray absorptiometry.

**Results:**

A total of 1,791 participants (10.3%) presented low muscle mass, and there was a sequential increase in the percentage of low muscle mass across remnant-C quartiles (Q1, 5.2%; Q2, 8.7%; Q3, 11.5%; Q4, 15.7%). In the full adjusted model, those in the highest remnant-C quartile group showed significantly increased odds ratio (OR) for low muscle mass compared with those in the lowest remnant-C group after adjusting for various confounding factors (OR = 1.33, 95% confidence interval (CI) = 1.06–1.68, *P <*0.05). A wide range of subgroups and sensitivity analyses showed consistent results, supporting the robustness of our findings.

**Conclusions:**

Increased remnant-C value was associated with a high risk of low muscle mass in the Korean population. Remnant-C may be a novel marker for the prediction and management of sarcopenia in aging societies.

## Introduction

Sarcopenia is characterized by the loss of skeletal muscle mass, lower muscle strength, and/or physical performance, which imposes a global health burden, leading to an increased risk of falls, functional decline, and mortality (1). The prevalence of sarcopenia is estimated to be 5%–13% in population aged 60–70 years, and 11%–50% in those aged 80 or above (2). It is projected to increase at an accelerated pace in aging societies. Despite its adverse health outcomes, it is difficult to manage sarcopenia in clinical practice, because there are currently no FDA-approved drugs targeting sarcopenia. Therefore, evaluating and managing traditional and novel risk factors is important to minimize the adverse health outcomes caused by sarcopenia.

Recently, remnant cholesterol (remnant-C) has drawn wide attention as a lipid profile based on the cholesterol content of remnant lipoprotein particles, which are derived by lipolysis from triglyceride-rich lipoproteins (TRLs), including chylomicron remnants, very-low-density lipoproteins, and intermediate-density lipoproteins (3). In spite of definitive role of low-density lipoprotein cholesterol (LDL-C) lowering, there is a substantial residual risk of atherosclerotic cardiovascular disease (ASCVD). Owing to its atherosclerotic characteristics, remnant-C has been proposed as a key parameter for assessing the residual risk of ASCVD beyond traditional lipid profiles (4). Furthermore, remnant-C may be more atherogenic than LDL-C because of its pro-inflammatory effects and persistent retention time in the vasculature (5). Several longitudinal studies have demonstrated a remarkable association between remnant-C and ASCVD, even after LDL-C reduction to the recommended target (4, 5). Moreover, other metabolic diseases such as type 2 diabetes mellitus, metabolic syndrome, and non-alcoholic fatty liver disease (NAFLD) have been reported to be associated with remnant-C in previous studies (6, 7). Therefore, new medications targeting remnant-C, such as novel inhibitors of Apo-CIII and angiopoietin-like 3 (ANGPLT3), have been actively explored (8).

Dyslipidemia can induce ectopic fat deposition, and lipid accumulation between and within muscle cells disturbs normal physiology, leading to decreased muscle mass and quality (9). Mitochondrial dysfunction, oxidative stress, and increased inflammatory responses through various mediators such as proinflammatory cytokines can explain this vicious relationship between lipids and muscles, although the exact molecular mechanism remains to be elucidated (9). Considering that skeletal muscle is involved in regulating lipid metabolism and remnant-C is linked to various metabolic disorders (10, 11), the association between remnant-C and sarcopenia might be an interesting research topic. Few studies have reported an association between sarcopenia/sarcopenic obesity and dyslipidemia as a component of metabolic syndrome (12, 13). However, limited studies have investigated the association between remnant-C and sarcopenia.

Therefore, this study aimed to identify the relationship between remnant-C and low muscle mass, a major factor defining sarcopenia, using nationwide population-based data.

## Methods

### Data source and study design

We used data from the Korean National Health and Nutrition Examination Surveys (KNHANES) between 2008 and 2011. The KNHANES is a nationwide cross-sectional survey regularly conducted by the Korean Center for Disease Control and Prevention to monitor the health and nutritional status of the general population in South Korea (14). Each KNHANES is comprised of independent datasets as previously demonstrated (15). The 4^th^ KNHANES was conducted from 2007 to 2009, and the 5^th^ survey was conducted from 2010 to 2012. We used data from 2^nd^ and 3^rd^ year of the 4^th^ KNANES (IV-2, IV-3) as well as 1^st^ and 2^nd^ year of the 5^th^ survey (V-1, V2) because they included assessments of muscle mass.

**Figure 1:**
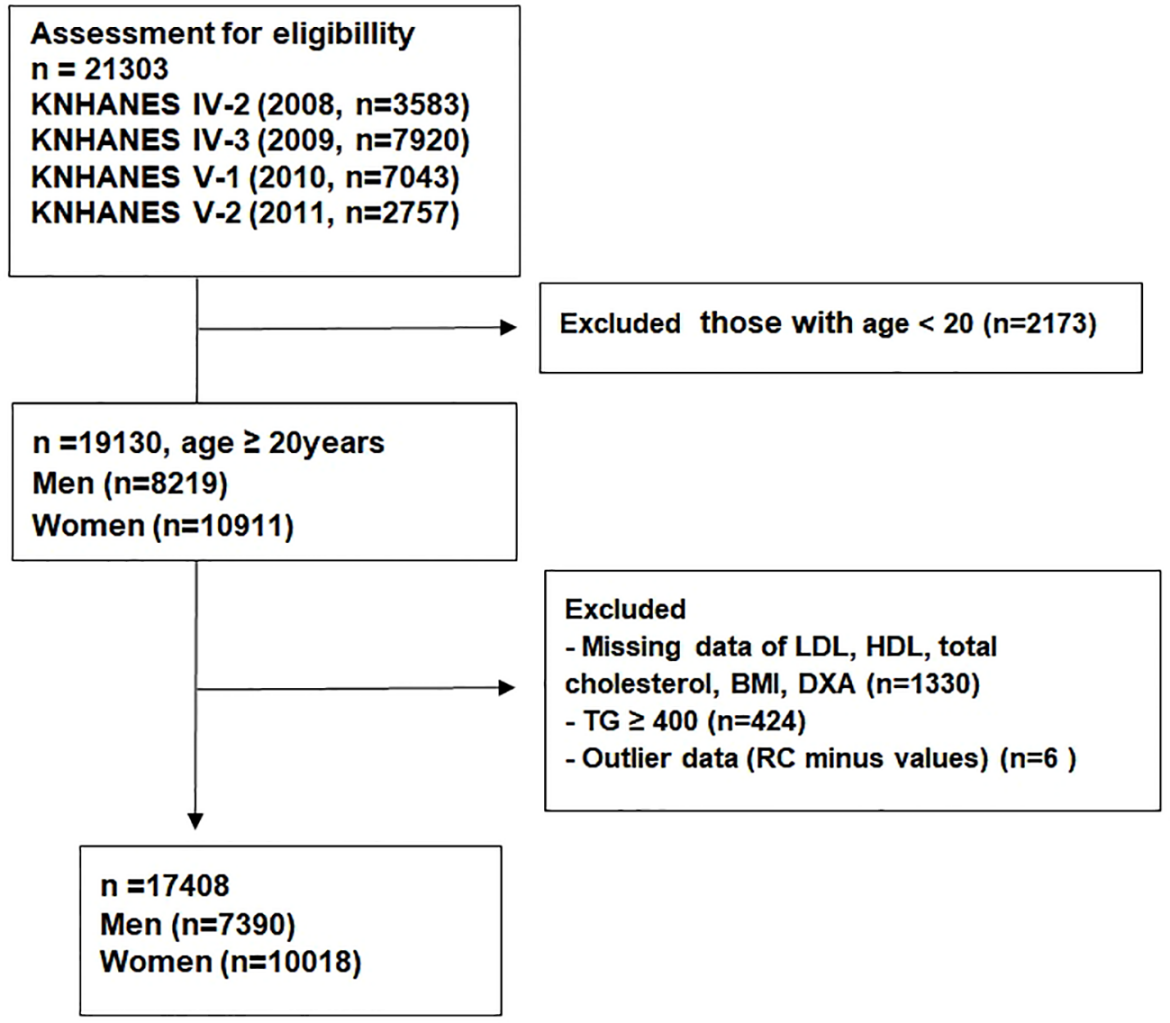
Inclusion and exclusion of study participants.

Among the 21,303 subjects from the KNHANES 2008–2011, participants aged <20 years (n = 2,173) were excluded. Subjects with TG level ≥400 mg/dL (n = 424) and missing data (n = 1,330) were also excluded. We considered remnant-C minus values (n = 6) as outlier data and excluded from the analysis. Ultimately, 17,408 participants were included in the analysis (7,390 men, 10,018 women; **Figure 1**).

This study was approved by the Institutional Review Board of Korea University and was conducted in accordance with the Declaration of Helsinki of the World Medical Association.

### Assessment of remnant cholesterol and definition of low muscle mass

Blood samples were collected after an overnight fast. Remnant-C was calculated as total cholesterol minus LDL-C and HDL-C. Appendicular skeletal muscle mass (ASM) was measured using dual-energy X-ray absorptiometry. Low muscle mass was defined as ASM (kg) divided by body mass index (BMI, kg/m^2^) <0.789 for men and <0.512 for women, according to The Foundation for the National Institutes of Health Sarcopenia Project (16).

### Covariates

Hypertension was defined as blood pressure ≥140/90 mmHg or the use of anti-hypertensive medication. Diabetes was defined as fasting blood glucose (FBG) ≥126 mg/dL or use of oral hypoglycemic agents or insulin. Dyslipidemia was defined as fasting total cholesterol ≥240 mg/dL or use of lipid lowering agents. Metabolic syndrome was defined according to the modified National Cholesterol Education Program criteria (17). Estimated glomerular filtration rate (eGFR) was calculated from Chronic Kidney Disease Epidemiology Collaboration equation (CKD-EPI) (18), and CKD was defined as eGFR ≤60 mL/min/1.73 m^2^. Homeostasis model assessment of insulin resistance (HOMA-IR) was calculated as fasting serum insulin (μIU/mL) × fasting glucose (mg/dL)/405. Smoking status was categorized as never smoked, ex-smoker, or current smoker. High risk drinking was defined as ≥7 cups for men, ≥5 cups for women at a time, and ≥2 times per week. Regular exercise was defined as moderate or high-intensity physical activity on a regular basis (≥3 times per week and ≥20 min at a time) (19). Household incomes were divided into quartiles.

### Statistical analysis

The subjects were divided into four groups according to the quartile of remnant-C values (6). The characteristics of the participants are expressed as mean ± standard deviation (SD) for continuous variables and as number (%) for categorical variables. Differences between groups were compared using Student’s t-test for continuous variables and the chi-square test for categorical variables. Complex sample analysis was conducted using unadjusted and adjusted multivariable logistic regression models to evaluate the association between remnant-C and low muscle mass. Subgroup analysis was performed and stratified by age, sex, BMI, LDL-C level, eGFR, FBG level, and systolic blood pressure (SBP). Additionally, a sensitivity analysis was performed to control for the effects of lipid-lowering agents and underlying diseases. *P* value <0.05 was considered statistically significant. All the analyses were performed using SAS version 9.4 (SAS Institute Inc., Cary, NC, USA).

## Results

### Baseline characteristics of the study population

The baseline characteristics of the study participants according to the remnant-C quartiles are summarized in **Table 1**. Participants in the higher remnant-C quartiles were more likely to be male and older than those in the lower remnant-C quartiles. They had higher BMI, waist circumference, blood pressure, FBG, HOMA-IR, total cholesterol, LDL-C, triglycerides (TG), and liver enzymes, as well as lower eGFR. The proportions of current smokers and heavy drinkers were higher in participants in high remnant-C quartile group, and they tended to exercise less regularly. The prevalence of coronary heart disease, cerebrovascular disease, hypertension, metabolic syndrome, diabetes, and hypercholesterolemia was also higher in participants in the high remnant-C quartiles.

**Table 1:** Baseline characteristics according to quartiles of remnant cholesterol.

	Q1 (N = 4351)	Q2 (N = 4353)	Q3 (N = 4352)	Q4 (N = 4352)	*P*-value
Remnant Cholesterol (mg/dL)	11.17 ± 2.47	17.93 ± 1.88	25.45 ± 2.66	43.78 ± 11.75	<0.001
Female [n (%)]	3017 (69.3)	2627 (60.3)	2346 (53.9)	2028 (46.6)	<0.001
Age (years)	43.31 ± 15.58	48.12 ± 15.96	51.55 ± 15.48	53.85 ± 14.48	<0.001
Body mass index (kg/m^2^)	22.24 ± 3.07	23.16 ± 3.21	23.98 ± 3.24	24.94 ± 3.09	<0.001
Waist circumference (cm)	76.09 ± 9.28	79.46 ± 9.43	82.39 ± 9.34	86.00 ± 8.59	<0.001
Systolic BP (mmHg)	111.18 ± 15.99	115.90 ± 17.12	119.34 ± 17.18	123.38 ± 17.23	<0.001
Diastolic BP (mmHg)	71.38 ± 9.99	73.90 ± 10.16	75.62 ± 10.17	78.11 ± 10.49	<0.001
Fasting glucose (mg/dL)	91.84 ± 15.43	94.93 ± 18.02	98.91 ± 22.53	104.11 ± 28.56	<0.001
Fasting insulin (µIU/mL)	8.63 ± 3.81	9.36 ± 4.27	10.10 ± 5.95	11.26 ± 6.05	<0.001
HOMA-IR	1.98 ± 1.11	2.22 ± 1.24	2.53 ± 2.41	2.94 ± 2.42	<0.001
Total cholesterol (mg/dL)	172.78 ± 30.99	181.80 ± 32.34	191.70 ± 33.72	202.67 ± 36.30	<0.001
HDL-C (mg/dL)	54.27 ± 11.03	49.93 ± 10.54	47.53 ± 10.63	42.52 ± 9.69	<0.001
LDL-C (mg/dL)	107.34 ± 28.00	113.94 ± 30.23	118.72 ± 32.09	116.37 ± 34.85	<0.001
Triglyceride (mg/dL)	57.98 ± 16.48	89.08 ± 19.85	125.71 ± 26.90	219.47 ± 64.01	<0.001
AST (U/L)	20.17 ± 10.49	21.13 ± 9.57	22.69 ± 13.64	24.74 ± 13.35	<0.001
ALT (U/L)	16.89 ± 12.31	19.19 ± 13.11	22.32 ± 24.53	26.42 ± 18.62	<0.001
BUN (mg/dL)	14.04 ± 4.24	14.27 ± 4.37	14.56 ± 4.84	14.59 ± 4.23	<0.001
Cr (mg/dL)	0.77 ± 0.16	0.81 ± 0.23	0.84 ± 0.26	0.86 ± 0.24	<0.001
eGFR (mL/min/1.73 m^2^)	105.51 ± 14.75	100.90 ± 15.77	97.47 ± 16.46	95.35 ± 16.24	<0.001
Smoking history [n (%)]					<0.001
Never smoker	3095 (71.6)	2793 (64.5)	2532 (58.5)	2190 (50.6)	
Ex-smoker	558 (12.9)	713 (16.5)	798 (18.4)	940 (21.7)	
Current smoker	671 (15.5)	822 (19.0)	997 (23.0)	1201 (27.7)	
High risk drinking [n (%)]	305 (7.1)	396 (9.2)	501 (11.6)	684 (15.8)	<0.001
Regular exercise [n (%)]	1120 (25.9)	1059 (24.5)	1025 (23.7)	990 (22.9)	0.00741
Coronary heart disease [n (%)]	64 (1.5)	102 (2.4)	120 (2.8)	128 (3.0)	<0.001
Cerebrovascular disease [n (%)]	41 (0.9)	74 (1.7)	105 (2.4)	110 (2.5)	<0.001
Hypertension [n (%)]	619 (14.3)	989 (22.8)	1350 (31.2)	1764 (40.7)	<0.001
Metabolic syndrome [n (%)]	279 (6.4)	617 (14.2)	1132 (26.0)	2915 (67.0)	<0.001
Diabetes mellitus [n (%)]	180 (4.3)	309 (7.3)	451 (10.7)	666 (15.9)	<0.001
Percentage (%) of low muscle mass	226 (5.2)	379 (8.7)	501 (11.5)	685 (15.7)	<0.001
Income [n (%)]					0.283
Q1	1065 (24.7)	1051 (24.5)	1030 (24.0)	1078 (25.1)	
Q2	1043 (24.2)	1078 (25.1)	1097 (25.6)	1138 (26.5)	
Q3	1110 (25.8)	1091 (25.4)	1076 (25.1)	1073 (25.0)	
Q4	1092 (25.3)	1075 (25.0)	1090 (25.4)	1006 (23.4)	
Dyslipidemia [n (%)]	191 (4.5)	365 (8.6)	589 (13.9)	884 (21.1)	<0.001

Values are presented as number (%), mean ± standard deviation.

BP, blood pressure; HOMA-IR, homeostasis model assessment of insulin resistance; HDL-C, high-density lipoprotein cholesterol; LDL-C, low-density lipoprotein cholesterol; AST, aspartate aminotransferase; ALT, alanine aminotransferase; eGFR, estimated glomerular filtration rate.

**Figure 2:**
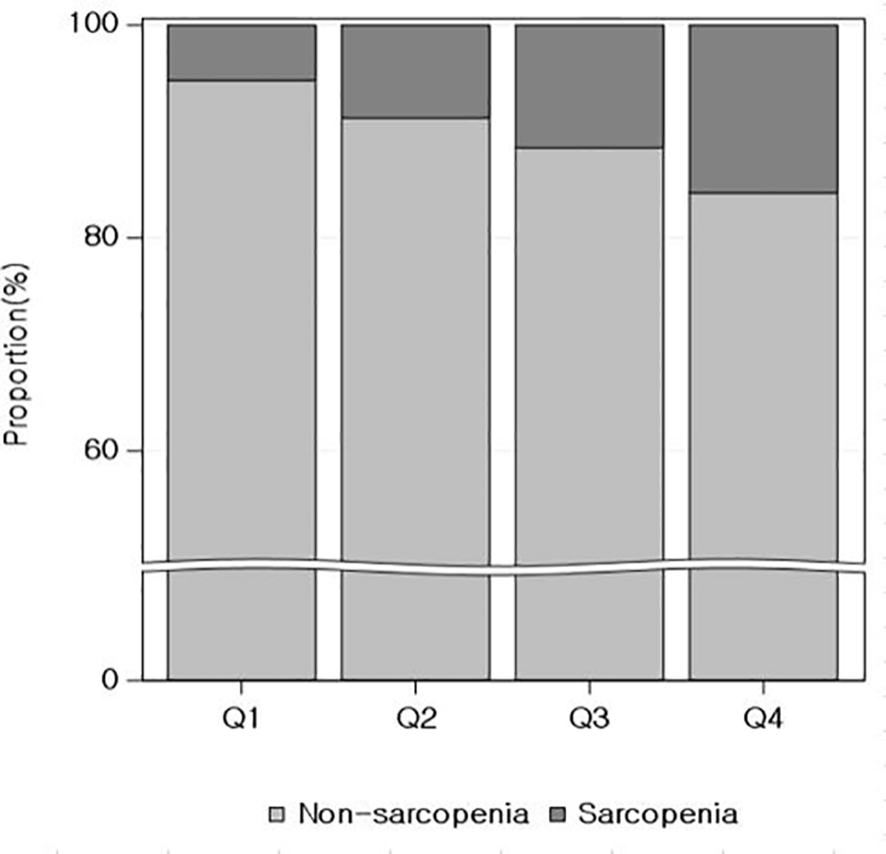
Relationship between quartiles of remnant cholesterol and the proportion of low muscle mass.

Of the 17,408 participants, 1,791 (10.3%) had low muscle mass. There was a sequential increase in the percentages of low muscle mass across the remnant-C quartiles from Q1 to Q4 [5.2%, 8.7%, 11.5%, and 15.7%, respectively (*P* for trend <0.01)] (**Figure 2**).

### Association between remnant-C and risk of low muscle mass

The odds ratios (ORs) and 95% confidence intervals (CIs) for low muscle mass according to remnant-C quartiles are presented in **Table 2**. The crude model exhibited sequential increases in ORs across remnant-C quartiles (OR [95% CI]: first quartile [Q1], reference; second quartile [Q2], 1.7 [1.38-2.09]; third quartile [Q3], 2.18 [1.8-2.65]; fourth quartile [Q4], 3.44 [2.84-4.17]). This sequential trend persisted after adjusting for age and sex (Q2, 1.31 [1.06-1.61]; Q3, 1.43 [1.17-1.74]; Q4, 2.05 [1.68-2.52]) (Model 1). After adjusting for age, sex, and BMI, the second quartile group showed a higher risk of low muscle mass than the third quartile group; however, the fourth quartile group still showed the highest risk among all groups. In the fully adjusted model, the trend persisted and statistical significance was maintained. The highest remnant-C quartile group presented a 33% increased risk of low muscle mass compared with the lowest quartile group (Model 6, Q4, 1.33 [1.06-1.68], *P <*0.05).

**Table 2:** Odds ratios for low muscle mass according to quartiles of remnant cholesterol.

	Crude model	Model 1	Model 2	Model 3	Model 4	Model 5	Model 6
Q1	1 (ref.)	1 (ref.)	1 (ref.)	1 (ref.)	1 (ref.)	1 (ref.)	1 (ref.)
Q2	1.7 (1.38,2.09)	1.31 (1.06,1.61)	1.13 (0.9,1.41)	1.14 (0.91,1.42)	1.13 (0.91,1.41)	1.13 (0.91,1.41)	1.13 (0.9,1.42)
Q3	2.18 (1.8,2.65)	1.43 (1.17,1.74)	1.04 (0.84,1.29)	1.06 (0.86,1.31)	1.07 (0.86,1.32)	1.07 (0.86,1.32)	1.07 (0.85,1.33)
Q4	3.44 (2.84,4.17)	2.05 (1.68,2.52)	1.32 (1.06,1.64)	1.31 (1.05,1.63)	1.32 (1.06,1.65)	1.33 (1.06,1.66)	1.33 (1.06,1.68)
*P*-value	<0.001	<0.001	0.022	0.041	0.034	0.031	0.034
*P* for trend	<0.001	<0.001	0.028	0.032	0.025	0.024	0.028

Model 1: adjusted for age and sex.

Model 2: adjusted for model 1 plus body mass index.

Model 3: adjusted for model 2 plus fasting glucose, estimated glomerular filtration rate, and systolic blood pressure.

Model 4: adjusted for model 3 plus dyslipidemia medication.

Model 5: adjusted for model 4 plus history of cerebrovascular and coronary heart disease.

Model 6: adjusted for model 5 plus drinking, smoking, exercise, and income.

### Subgroup analysis

**Figure 3:**
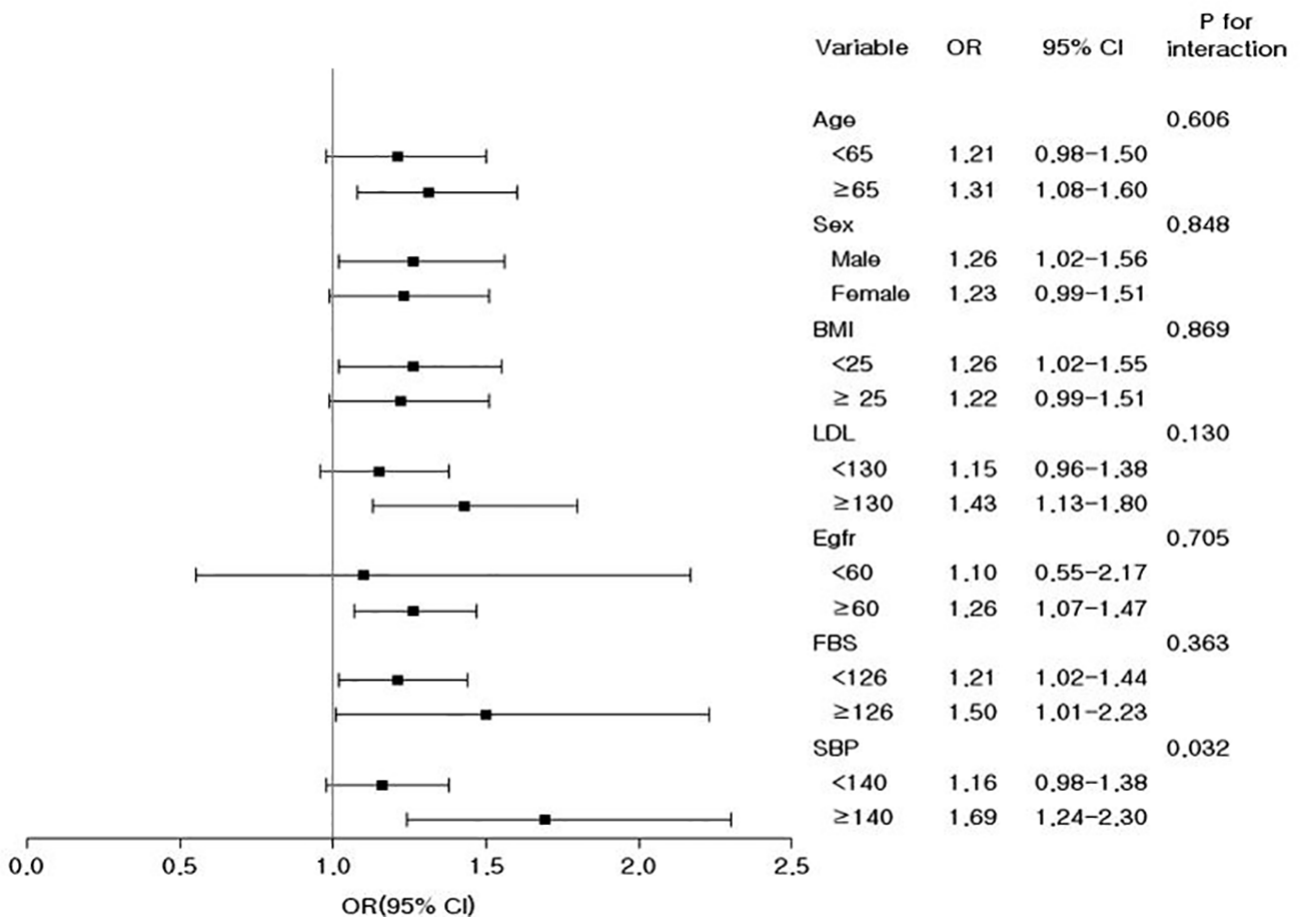
Odds ratios and 95% confidence intervals for the prevalence of low muscle mass in the highest quartile versus the remaining three quartiles of remnant cholesterol in the subgroups. Adjusted for age, sex, body mass index, fasting glucose, estimated glomerular filtration rate, systolic blood pressure, dyslipidemia medication, history of cerebrovascular and coronary heart disease, drinking, smoking, exercise, and income.

Stratification by age, sex, BMI, LDL-C, eGFR, FBG, and SBP was performed and the relationship between remnant-C and low muscle mass was investigated (**Figure 3**). In all subgroups, the highest quartile group (Q4) of remnant-C had an increased risk of low muscle mass compared with the rest three quartile groups (Q1-Q3). In hypertensive patients, the association between remnant-C and increased risk of low muscle mass was more remarkable than those without hypertension, implicating the synergistic effect of hypertension and dyslipidemia on muscle (*P* for interaction <0.05). However, age, sex, BMI, LDL-C, eGFR, and FBS did not significantly modify the effect.

### Sensitivity analysis

To reinforce our results, we conducted a sensitivity analysis (**Table 3**). As lipid-lowering agents can significantly influence blood lipid profiles, we analyzed the participants after excluding those using dyslipidemia medication and confirmed results consistent with the main analysis (Model 6: Q4, 1.31 [1.03-1.67], *P <*0.05). Furthermore, to clarify the independent effect of remnant-C on low muscle mass, regardless of underlying diseases, we excluded subjects with a history of coronary syndrome, stroke, diabetes, and CKD, respectively. The results corresponded with those of the main analysis, and statistical significance was maintained in all models.

**Table 3:** Odds ratios and 95% confidence intervals for low muscle mass according to medication and underlying diseases.

	Crude model	Model 1	Model 2	Model 3	Model 4	Model 5	Model 6
After excluding those on dyslipidemia medication							
Q1	1 (ref.)	1 (ref.)	1 (ref.)	1 (ref.)	–	1 (ref.)	1 (ref.)
Q2	1.69 (1.36,2.09)	1.3 (1.04,1.61)	1.13 (0.9,1.41)	1.14 (0.91,1.43)	-	1.14 (0.91,1.43)	1.13 (0.9,1.43)
Q3	2.11 (1.73,2.58)	1.39 (1.14,1.71)	1.04 (0.83,1.29)	1.06 (0.85,1.32)	-	1.06 (0.85,1.32)	1.05 (0.84,1.33)
Q4	3.4 (2.78,4.15)	2.02 (1.64,2.5)	1.31 (1.05,1.65)	1.31 (1.04,1.65)	-	1.31 (1.04,1.65)	1.31 (1.03,1.67)
*P*-value	<0.001	<0.001	0.036	0.061	-	0.061	0.065
*P* for trend	<0.001	<0.001	0.041	0.042	-	0.042	0.052
After excluding those with history of coronary heart disease							
Q1	1 (ref.)	1 (ref.)	1 (ref.)	1 (ref.)	1 (ref.)	–	1 (ref.)
Q2	1.67 (1.35,2.07)	1.29 (1.04,1.6)	1.11 (0.88,1.39)	1.12 (0.89,1.41)	1.12 (0.89,1.41)	-	1.12 (0.89,1.42)
Q3	2.19 (1.79,2.68)	1.45 (1.18,1.78)	1.06 (0.85,1.32)	1.08 (0.87,1.35)	1.08 (0.87,1.35)	-	1.08 (0.86,1.36)
Q4	3.47 (2.85,4.22)	2.08 (1.69,2.55)	1.33 (1.06,1.66)	1.32 (1.06,1.66)	1.33 (1.06,1.66)	-	1.34 (1.06,1.69)
*P*-value	<0.001	<0.001	0.023	0.044	0.042	-	0.041
*P* for trend	<0.001	<0.001	0.023	0.024	0.023	-	0.025
After excluding those with history of stroke							
Q1	1 (ref.)	1 (ref.)	1 (ref.)	1 (ref.)	1 (ref.)	–	1 (ref.)
Q2	1.68 (1.36,2.07)	1.30 (1.05,1.61)	1.11 (0.88,1.39)	1.12 (0.89,1.40)	1.12 (0.89,1.40)	-	1.11 (0.88,1.40)
Q3	2.19 (1.80,2.67)	1.46 (1.19,1.78)	1.06 (0.86,1.32)	1.08 (0.87,1.34)	1.09 (0.87,1.35)	-	1.08 (0.86,1.35)
Q4	3.49 (2.87,4.25)	2.12 (1.72,2.61)	1.35 (1.08,1.69)	1.35 (1.08,1.69)	1.35 (1.08,1.70)	-	1.35 (1.07,1.70)
*P*-value	<0.001	<0.001	0.015	0.026	0.025	-	0.033
*P* for trend	<0.001	<0.001	0.014	0.015	0.014	-	0.020
After excluding those with history of diabetes							
Q1	1 (ref.)	1 (ref.)	1 (ref.)	1 (ref.)	1 (ref.)	1 (ref.)	1 (ref.)
Q2	1.70 (1.36,2.13)	1.34 (1.07,1.68)	1.17 (0.93,1.48)	1.19 (0.94,1.50)	1.19 (0.94,1.50)	1.19 (0.94,1.50)	1.19 (0.93,1.51)
Q3	2.16 (1.76,2.66)	1.48 (1.20,1.83)	1.11 (0.88,1.39)	1.15 (0.92,1.45)	1.15 (0.92,1.45)	1.15 (0.91,1.45)	1.15 (0.91,1.46)
Q4	3.50 (2.81,4.36)	2.20 (1.74,2.77)	1.43 (1.11,1.83)	1.48 (1.15,1.90)	1.48 (1.15,1.89)	1.48 (1.15,1.89)	1.48 (1.15,1.92)
*P*-value	<0.001	<0.001	0.026	0.014	0.014	0.015	0.017
*P* for trend	<0.001	<0.001	0.008	0.003	0.003	0.004	0.004
After excluding those with chronic kidney disease							
Q1	1 (ref.)	1 (ref.)	1 (ref.)	1 (ref.)	1 (ref.)	1 (ref.)	1 (ref.)
Q2	1.62 (1.31,1.99)	1.26 (1.02,1.56)	1.08 (0.87,1.35)	1.1 (0.88,1.38)	1.09 (0.87,1.37)	1.09 (0.87,1.37)	1.08 (0.86,1.36)
Q3	2.1 (1.73,2.55)	1.41 (1.16,1.72)	1.03 (0.83,1.28)	1.08 (0.87,1.33)	1.08 (0.87,1.34)	1.08 (0.87,1.34)	1.08 (0.87,1.36)
Q4	3.31 (2.72,4.02)	2.04 (1.66,2.5)	1.3 (1.04,1.62)	1.32 (1.05,1.65)	1.33 (1.06,1.67)	1.33 (1.06,1.67)	1.34 (1.06,1.69)
*P*-value	<0.001	<0.001	0.039	0.052	0.039	0.037	0.040
*P* for trend	<0.001	<0.001	0.036	0.023	0.017	0.017	0.019

Model 1: adjusted for age and sex.

Model 2: adjusted for model 1 plus body mass index.

Model 3: adjusted for model 2 plus fasting glucose, estimated glomerular filtration rate, and systolic blood pressure.

Model 4: adjusted for model 3 plus dyslipidemia medication.

Model 5: adjusted for model 4 plus history of cerebrovascular and coronary heart disease.

Model 6: adjusted for model 5 plus drinking, smoking, exercise, and income.

Exclusion criteria cannot be adjusted, so they are indicated as – in each model.

## Discussion

The present study showed that Korean adults with elevated levels of remnant-C demonstrated a high prevalence of low muscle mass. These findings remained consistent even after adjusting for potential confounding factors in our large national population-based database. The present results suggest that patients with increased remnant-C may be needed to evaluate sarcopenia and its associated comorbidities.

Changes in body composition, such as redistribution of muscle and fat, are characteristic during human aging. Decreased muscle mass can result in loss of independence in everyday life and exacerbate diseases of vital organs, leading to high mortality via signaling mediators called myokines (9). Therefore, the global burden and importance of sarcopenia are inevitably increasing in a progressive aging society. Currently, the interventions for preventing and treating sarcopenia focus on physical exercise and protein supplementations (20). However, the enforcement of resistance exercises with proper nutritional support for the elderly is challenging because of anabolic resistance and poor long-term compliance (20, 21). Therefore, investigating novel prophylactic and therapeutic targets for sarcopenia is required.

Several epidemiological studies and clinical trials have shown that remnant-C predicts the risk of ASCVD, which may surpass LDL-C and ApoB levels (22). On a per particle basis, remnant particles have up to four-fold greater cholesterol than LDL-C particles owing to their larger size (23). By including remnant-C levels in a conventional risk model, the reclassification of individuals to predict future cardiovascular events is improved in primary and secondary prevention settings (24). Remnant-C has attracted attention as a residual risk factor for ASCVD because it is permeable to the arterial intima and is retained in the arterial wall, making it atherogenic (25). The accumulated remnant-C is absorbed by macrophages and smooth muscle cells, resulting in the formation of foam cells and finally develop atherosclerotic plaques (4, 26). Skeletal muscle is highly vascularized, and vascular system from feeding arteries branching into arterioles and capillaries play a significant role in muscle metabolism, mainly through transporting nutrition and oxygen (27). Remnants of TRLs may easily cross the endothelium in the vasculature of skeletal muscle. Accumulation of remnant-C would facilitate foam cell formation, leading to atherosclerotic plaques. Thickened blood vessel walls and narrowed vessels might hamper sufficient nutrition and oxygen supply to skeletal muscle, which is critical in muscle growth and function.

Few studies have evaluated the relationship between dyslipidemia and sarcopenia/sarcopenic obesity. Lu et al. reported a 12-fold increase in the risk of metabolic syndrome in patients with sarcopenic obesity compared to the normal group (13). As components of the metabolic syndrome, there is a significant association between sarcopenic obesity and serum triglyceride and HDL-C levels (13). Baek et al. examined dyslipidemia as a separate factor associated with sarcopenia/sarcopenic obesity in the elderly Korean population (12). Bi and Dong et al. showed that dyslipidemia is positively related with sarcopenia in the elderly population, and this association was significantly affected by age, sex, and location of the participants (28). Vella et al. reported that abdominal muscle area was inversely correlated with total cholesterol, triglycerides, and very low density cholesterol (29). In addition, plasma HDL-C level has been reported as a meaningful predictor of sarcopenia in healthy old people (30). Gong et al. reported that the levels of TRLs and their remnants were higher in the sarcopenia group than in the control group in 84 elderly Chinese subjects, using bioelectrical impedance analysis (BIA) to assess skeletal muscle mass (10). With established cardiovascular benefits of lipid lowering drugs, such as statins, there is still a controversy on the association between lipid lowering drugs and sarcopenia (31). Further research needs to be done to elucidate effects of dyslipidemia medications on muscle. Our results underscore the relationship between dyslipidemia and sarcopenia, which imposes a critical burden on future aging societies. In subgroup analysis, the relationship between remnant-C and low muscle mass was more prominent in hypertensive patients. Although there are some previous studies demonstrating that hypertension is associated with increased risk of sarcopenia (32), there is still a controversy about the impact of hypertension on sarcopenia (33). Whether comorbidity of dyslipidemia and hypertension confers synergistic effect on muscle health is yet to be elucidated. Nevertheless, possible link between ischemic heart disease and frailty, whose characteristics share common factors with sarcopenia, has been raised (34). More comprehensive future research in clinical and biological fields is required to clarify the interconnection among dyslipidemia, cardiovascular disease, and muscle disease.

The underlying mechanism linking remnant-C to low muscle mass is not yet fully understood. Saturated fatty acids such as palmitate induce muscle atrophy by modulating insulin signaling. Palmitate reduces Akt activity and increases nuclear FoxO3 protein levels, which leads to increased expression of atrophy-inducing genes (atrogenes), such as the E3 ubiquitin ligase atrogin-1/MAFbx and the autophagy mediator Bnip3 (35). Palmitate treatment increases intramyocellular lipids and induces myotube atrophy (36). The accumulation of lipids and their derivatives, such as diacylglycerol and ceramides, has been reported to be toxic to muscles. One of their harmful effects is insulin resistance, which is a pivotal pathophysiological factor in sarcopenia. Previous research has indicated that the deposition of free fatty acids in muscles disrupts insulin signaling, leading to insulin resistance (37). TG involved in TRLs are hydrolyzed by lipoprotein lipase on the surface of the capillary endothelium in skeletal muscles (23), producing fatty acids as metabolites that reduce insulin sensitivity. Grice et al. reported that excessive cholesterol accumulation in skeletal muscle membranes is an early reversible aspect of insulin resistance in high fat diet-fed mice (38). Cholesterol accumulation within cells can activate RhoA, which is significantly involved in cellular functions, and damage them in the liver and heart (39, 40). Given that RhoA is important in the differentiation and proliferation of skeletal muscle cells, the accumulation of cholesterol itself might disturb the normal physiology of skeletal muscle, such as by balancing protein anabolism/catabolism or maintaining glucose homeostasis. Excessive remnants could contribute to ectopic fat deposition in skeletal muscle and lead to sarcopenic state, although whether and how specific composition of lipids have different effects on muscle is not fully identified.

As remnant-C has been reported to be associated with low-grade inflammation (41), inflammatory responses may explain the association between remnant-C and sarcopenia. Unlike LDL-C, remnant-C which passed through the vessel wall easily forms foam cell because remnant-C needs no modification to be trapped by macrophage (42). Foam cell is a well-known attribute of inflammation (43). Remnant-C could induce pro-inflammatory cytokines such as tumor necrosis factor-α and interleukine-1, 6, 8 (44), and skeletal muscle react to pro-inflammatory cytokines with muscle atrophy (9). Pro-inflammatory cytokines may antagonize the anabolism of insulin-like growth factor-1 (IGF-1) in muscle and promote cell apoptosis, which result in decreased muscle mass (45). Accumulating evidence suggests that sarcopenia is closely associated with chronic low-grade inflammation (46).

Increased oxidative stress and reactive oxygen species production have been reported to be associated with increased TRLs and their lipolysis (47). Remnant-C levels are inversely correlated with HDL-C levels, which could prevent oxidative stress (22). Furthermore, TRLs lipolysis elicits endothelial cell inflammation and dysfunction by stimulating the production of reactive oxygen species (48). Excessive oxidative stress and the production of reactive oxygen species induce protein misfolding and reduce protein synthesis (37), which may contribute to low muscle mass.

This study had several limitations. First, this is a cross-sectional study; therefore, the causal relationship between remnant-C and sarcopenia cannot be elucidated. Secondly, although low muscle mass is an important component of sarcopenia, the lack of information on muscle strength and physical performance in the present database does not fully explain the relationship between remnant-C and sarcopenia. Future studies including assessments of muscle strength and physical performance would be needed to clarify the relationship between remnant-C and sarcopenia. Third, there was no detailed information on the dyslipidemia medications. However, we analyzed the participants after excluding those taking lipid-lowering agents and confirmed consistent results. Fourth, although we have excluded confounding effects of lipid-associated diseases including CVD, diabetes, or CKD, we could not exclude muscle diseases such as Duchenne muscular dystrophy due to lack of detailed information. The effect of muscle-associated diseases should be addressed for more validated results in the future. Finally, directly measured remnant-C levels are not available in the KNHANES database. However, a relatively high concordance between measured and calculated remnant-C values was reported in a previous study (49). Furthermore, individuals with increased calculated remnant-C levels are at an increased risk of incident CVD independent of LDL-C levels in Korean population-based studies (50).

The strength of this study is that it is the first to show an association between remnant-C and low muscle mass using a validated nationwide survey with a large sample size, standard data collection methods, and the availability of a large number of confounding factors. Moreover, the results were confirmed by a wide range of subgroup and sensitivity analyses, excluding the confounding effects of underlying diseases, such as CVD, diabetes, and CKD.

## Conclusion

In conclusion, remnant-C is associated with low muscle mass in the Korean population and may be a promising marker for predicting and managing sarcopenia. Our results provide new insights into the impact of remnant-C on skeletal muscle in an aging society. Further research is needed to better understand the molecular mechanism linking remnant cholesterol and skeletal muscle and to support its clinical usefulness.

## Data Availability

The datasets presented in this study can be found in online repositories. The names of the repository/repositories and accession number(s) can be found in the article/supplementary material.
